# Spotlight on early-career researchers

**DOI:** 10.1038/s42003-018-0061-3

**Published:** 2018-06-25

## Abstract

Today we publish a Q&A with Dr. Marie Heffern, the first in a series of short interviews with early career researchers. We will be publishing these throughout the next year and welcome suggestions for featured researchers.

Early career researchers face fierce competition for funding and intense pressure to publish. They also represent the future of science. The challenges faced by early career researchers—young tenure-track investigators and postdocs—have received necessary attention in recent years, including in a special issue in *Nature* in 2016 (https://go.nature.com/2Koj47q). Despite these challenges, young investigators continue to make important and significant scientific contributions that deserve recognition. Many journals do recognize the achievements of early career researchers in multiple formats, including researcher profiles. However, this is one case where we believe there can not be too much of a good thing. Over the next 12 months or so in *Communications Biology*, we will highlight some of the rising stars among the many brilliant biologists around the world who are on their way to establishing their careers as independent researchers. Our first in this series, a Q&A with Dr. Marie Heffern, is published today^1^.

We hope that these profiles will inspire our readers to learn more about these researchers’ science and that they will provide valuable insight to younger scientists still in training.

At *Communications Biology*, we are committed to engaging researchers of all career stages in the publication process—from our editorial board, to our authors and reviewers. We are proud of our track record so far: of our published papers (as of 3rd May), about 28% have early career researchers (usually Assistant Professors or equivalent) as the most senior corresponding author. Nearly a third of our reviewers are early career researchers (31% of whom are postdocs) and a small percentage (~2%) are senior graduate students who were either recommended by their advisors or selected based on their publication record. We will continue to actively seek out the expertise of young investigators as well as more established scientists to ensure that we have an editorial perspective grounded in the realities facing our authors and readers.

We are aware that visibility of early career researchers is important for their career advancement. We believe that these researchers and their accomplishments should be known to the world and that they should receive more opportunities to contribute to the review process, speak at important conferences, and publish in high-profile journals. Although our series of short interviews with early career researchers is only a small effort toward this overall goal, we hope that these profiles will inspire our readers to learn more about these researchers’ science and that they will provide valuable insight to younger scientists still in training. Our in-house editors will select researchers to profile from among our most outstanding authors and reviewers, speakers at conferences we attend, and recommendations from readers or editorial board members. We welcome nominations (including self-nominations). If you or someone you know is an early career researcher who should be profiled in our pages, please contact us at commsbio@nature.com with your recommendation.

## References

[1] Spotlight on early career researchers: an interview with Marie Heffern. *Commun. Biol*. 10.1038/s42003-018-0057-z (2018). 10.1038/s42003-018-0057-z PMC6123709 30271958

